# Analysis of single nucleotide polymorphisms in chronic beryllium disease

**DOI:** 10.1186/s12931-021-01691-2

**Published:** 2021-04-16

**Authors:** Björn C. Frye, Karoline I. Gaede, Cesare Saltini, Milton D. Rossman, Dimitri S. Monos, Ken D. Rosenman, Christine R. Schuler, Ainsley Weston, Ralf Wegner, Rainer Noth, Gernot Zissel, Stefan Schreiber, Michael Nothnagel, Joachim Müller-Quernheim

**Affiliations:** 1 Department of Pneumology, Faculty of Medicine, Medical Center-University of Freiburg, University of Freiburg, Killianstrasse 5, 79106 Freiburg, Germany; 2 BioMaterial Bank Nord, Research Center Borstel, Leibniz Lung Center, 23845 Borstel, Parkallee 35 Germany; 3 Department of Medicine, University of Florida, 1600 Archer Rd, Gainesville, 32610 FL USA; 4 Pulmonary, Allergy, and Critical Care Division, University of Pennsylvania Medical Center, Philadelphia, USA; 5 Division of Occupational and Environmental Medicine, Department of Medicine, Michigan State University, East Lansing, MI USA; 6 Centers for Disease Control and Prevention, National Institute for Occupational Safety and Health, Morgantown, WV USA; 7 Institute for Occupational and Maritime Medicine, University Medical Center Hamburg-Eppendorf, Hamburg, Germany; 8 Institute for Clinical Molecular Biology, Christian-Albrechts University, Kiel, Germany; 9 Department of Statistical Genetics and Bioinformatics, Cologne Center for Genomics (CCG), University of Cologne, Cologne, Germany; 10 University Hospital Cologne, Cologne, Germany; 11 Airway Research Center North, German Center for Lung Research (DZL), Wöhrendamm 80, 22927 Großhansdorf, Germany

**Keywords:** Sarcoidosis, Berylliosis chronic, Beryllium diesase, Genetic, Annexin A11, BTNL2

## Abstract

Sarcoidosis and chronic beryllium disease (CBD) are phenocopies, however the latter one has a clear trigger factor that is beryllium exposure. This study analyses single nucleotide polymorphisms (SNPs) in a large cohort for beryllium-exposed persons. SNPs were chosen for their relevance in sarcoidosis. Even though one of largest cohorts of beryllium-exposed persons was analysed, no statistically relevant association between any SNP and CBD could be verified. Notably, some SNPs exhibit inverse OR for beryllium sensitization and CBD with nominally statistical significance, which allows hypothesizing about pathophysiological role of genes for the disease triggering and development.

Chronic beryllium disease (CBD) and sarcoidosis are granulomatous diseases similar in clinical presentation but different in their etiology [1]. Beryllium is the known trigger for CBD that elicits a type IV immune reaction with CD4 + (cluster of differentiation 4-positive) T-cell activation and proliferation (beryllium sensitization (BeS)). Beryllium sensitization (BeS) can be measured by the beryllium-specific lymphocyte proliferation test (BeLPT) allowing the differentiation between sarcoidosis and CBD [2]. A single SNP (single nucleotide polymorphism) in the HLA-DP (human leucocyte antigen of major histocompability complex II) represents a strong risk factor for disease development [3], but other genetic factors may contribute to beryllium sensitization and CBD. We hypothesized that SNPs described in sarcoidosis [4] may also be relevant for CBD and investigated SNPs from known sarcoidosis susceptibility loci (Interleukin 23 (*IL23)*, butyrophilin-like 2 (*BTNL2)*, ras-related protein 23 (rab23), Annexin A11 (ANXA11) and osteosarcoma 9 (OS9), in 1150 beryllium exposed, predominantly male individuals. These individuals were recruited from surveillance programs and outpatient clinics in US and Germany, including only individuals from Caucasian ancestry to reduce genetic heterogeneity. Beryllium exposure was assumed if the individual worked in a known beryllium-handling facility (thereby participating in surveillance programs) or a detailed occupational history revealed a workplace with a high susceptibility of beryllium exposure [2]. All participants had provided informed consent for their source studies. Within the studied cohort, 186 individuals had a confirmed abnormal BeLPT from peripheral blood and/or bronchoalveolar lavage. Of these individuals, thorough work-up including lung function, laboratory, radiological and histological investigations identified 93 individuals with granulomatous disease being classified as CBD individuals. In further 93 individuals no signs of granulomatous disease were found and they were considered as beryllium sensitized (93 individuals, BeS).

Eleven SNPs in five genes (Table 1 and *BTNL2* rs28362676 [4], the latter one being of low genotyping quality requiring cautious interpretation) were genotyped as described previously [5]. Statistical analyses were performed in different combinations (Table 1, nominal p-values and ORs). Calculations of ORs, confidence intervals and allele-based statistical analysis of genotype data using a chi-squared test (CQT), with Yates correction where applicable, and logistic regression were carried out in R v3.6 [6].

**Table 1:** Genetic association analysis was performed for beryllium-exposed individuals without disease (BeEx), beryllium sensitization (BeS), or diagnosed chronic beryllium disease (CBD) using a candidate gene approach

		*IL23*	*BTNL2*			*RAB23*			*ANXA11*		*OS9*
		rs11209026 **A**/G	rs28362677 **C**/T	rs28362678 **A**/G	rs2076530 **A**/G	rs772356421 **G**/A	rs11398 **T**/C	rs1040461 **T/**C	rs2573346 **A**/G	rs1049550 **A**/G	rs1050045 **C**/T
Amino acid change		R381/Q	M380I	P379L	S360G	–	–	G207S	–	R230C	-
Case	Control										
BeS	BeEx	0.730 (0.331–1.278), p = 0.443	1.191 (0.776–1.780), p = 0.478	1.020 (0.663–1.629), p = 1.000	1.214 (0.889–1.670), p = 0.258	1.037 (0.690–1.608), p = 0.952	0.950 (0.619–1.413), p = 0.887	1.165 (0.649–1.948), p = 0.681	1.271 (0.941–1.719), p = 0.134	*1.354* (1.002–1.829), p = 0.055*	1.126 (0.830–1.528), p = 0.490
CBD	BeEx	0.969 (0.495–1.704), p = 1.000	1.179 (0.765–1.767), p = 0.517	0.912 (0.600–1.431), p = 0.767	0.770 (0.566–1.048), p = 0.113	1.143 (0.757–1.790), p = 0.615	0.877 (0.567–1.310), p = 0.603	1.165 (0.649–1.948), p = 0.681	0.858 (0.627–1.166), p = 0.372	0.952 (0.696–1.293), p = 0.815	1.203 (0.883–1.640), p = 0.272
CBD	BeS	1.3456 (0.543–3.513), p = 0.675	0.987 (0.696–1.696), p = 1.000	0.899 (0.498–1.612), p = 0.834	*0.648* *(0.425–0.977), p = 0.044*	1.103 (0.622–1.967), p = 0.850	0.922 (0.524–1.615), p = 0.886	1.000 (0.455–2.200), p = 1.000	**0.712** **(0.482–1.044), p = 0.075**	0.736 (0.499–1.079), p = 0.112	1.072 (0.700–1.645), p = 0.836
CBD + BeS	BeEx	0.837 (0.5056–1.37), p = 0.561	1.184 (0.866–1.599), p = 0.325	0.964 (0.702- 1.343), p = 0.890	0.965 (0.769–1.212), p = 0.800	1.089 (0.802–1.500), p = 0.654	0.913 (0.668–1.229), p = 0.607	1.167 (0.764–1.730), p = 0.518	1.048 (0.838–1.308), p = 0.722	1.137 (0.910–1.42), p = 0.275	1.164 (0.928–1.461), p = 0.208
CBD	BeEx + BeS	0.991 (0.509–1.736), p = 1.000	1.157 (0.755–1.734), p = 0.564	0.912 (0.603–1.425), p = 0.760	**0.759** **(0.560–1.030), p = 0.089**	1.141 (0.755–1.786), p = 0.623	0.8789 (0.568–1.315), p = 0.616	1.150 (0.640–1.924), p = 0.717	0.842 (0.618–1.140), p = 0.300	0.927 (0.681–1.255), p = 0.680	1.191 (0.874–1.623), p = 0.300

Interleukin 23 (*IL23*, one SNP), *BTNL2* (four SNPs), ras-related protein 23 (*rab23*, three SNPs), Annexin A11 (*ANXA11*, two SNPs) and osteosarcoma 9 (*OS9*) were analyzed. Nominal P-values from an allele-based chi-squared test as well as the odds ratio, including 95% confidence intervals in parentheses, are shown. * p = 0.049 from an allele-based logistic regression. No results remain statistically significant after correction for multiple testing. Analysis for *BTNL2* rs28362676 were omitted from the table because unclear genotyping quality. Blod values indicate SNO with a nominal significance of p < 0.1. Italic values indicate SNP with a nominal significance of p < 0.05

None of the tested SNPs associated significantly with BeS or CBD after correction for multiple testing, most likely due to low numbers of BeS and CBD individuals. Misclassification of individuals to one group and genetic heterogeneity may be additional factors, even though cohort selection and experienced pneumological work-up should reduce this risk.

Although potentially explained by chance, the nominally significant OR for BTNL-2 and ANXA11 may allow some detailed hypothesizing on the role of these genes in CBD development, especially considering the fact that CBD (in contrast to sarcoidosis) develops in individuals with a known trigger via an intermediate step of beryllium sensitization (Fig. 1).

**Fig. 1:**
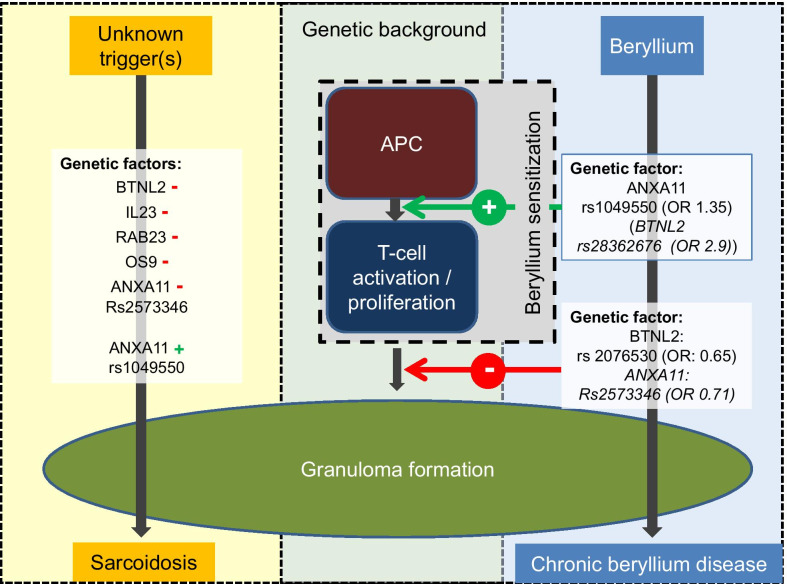
Sarcoidosis and chronic beryllium disease (CBD) share granuloma formation and clinical phenotype as common hallmarks. Genetic studies propose specific SNPs (e.g. in the *BTNL2* and *ANXA11* genes) contributing to disease development. Red—indicating enhanced susceptibility and green + indicating protection in sarcoidosis. In CBD, these SNPs influence disease initiation and progression differentially, which has not been studied so far in sarcoidosis. (APC: antigen presenting cells)

ANXA11 SNPs (rs2573346, rs1049550) associate differentially as risk or protection factor for sarcoidosis [7] and the nominal p-values in our tests suggested a similar association of ANXA11 in CBD. SNP rs1049550 (**A**/G) was associated with BeS (OR 1.35, Table 1, bold square, p = 0.055; p = 0.049 in logistic regression test). Slight discrepancies of CIs and p-values may result from imminent test conditions. SNP rs2573346 (**A**/G) showed borderline nominal significance for association with protection against CBD in BeS (OR 0.71, Table 1, dotted square). In summary, these results hint towards a role of ANXA in CBD, which may be comparable to the hypothesized function of ANXA 11. Interestingly, the effects might be linked to different steps during the development of CBD.

While BTNL-2 SNP rs2076530 (**A**/G) is a risk factor for sarcoidosis, it seems to protect individuals with BeS from developing CBD (OR 0.65 for CBD vs BeS, Table 1, bold square). Of note, this SNP has not been described for CBD, only the SNP rs3117099 within the BTNL-2 gene has been described to confer to CBD [8]. BTNL-2 SNP rs28362676 would have increased the risk of BeS (OR 2.9; data not shown), but low genotyping quality hindered its interpretation (therefore omitted from Table 1). Therefore this study could not demonstrate that BTNL2 SNPs were significantly associated with increased risk of BeS, but it might reduce the risk of CBD which fits well with the hypothesized function of BTNL2 in granulomatous disease by limiting a T-cell response [9]. This would attribute a dual role to BTNL2 in disease initiation and progression (similar to annexin A11, Fig. 1).

Even though this study analyzed one of the largest CBD cohorts, the overall number of BeS and CBD individuals was low for a genetic study, which limits the statistical power and interpretability. Still a cohort with 3 times more CBD patients would only be sufficiently powered to obtain significant results for very common risk alleles, i.e. with frequencies > 0.25, and with effect sizes stronger than those observed in our cohort, i.e. ORs > 2.0 (GAS Power Calculator; https://csg.sph.umich.edu/abecasis/gas_power_calculator/).

Despite this inherent limitation of genetic studies in rare diseases, these results allow hypothesizing on the pathogenic roles of some genes in granulomatous diseases. As depicted in Fig. 1, granuloma formation is the common final pathway of sarcoidosis and CBD. In difference to sarcoidosis, in CBD a unique initiating trigger is well defined and BeS is considered to be an intermediate step between beryllium exposure and CBD. The genetic analyses of BTNL-2 and ANXA 11 SNP point towards janiform roles of these genes in CBD with different effects on BeS and CBD. Different SNPs within these genes might associate with disease initiation or inversely with disease manifestation. As for sarcoidosis the intermediate step of T-cell activation without overt disease has not been characterized (Fig. 1), different genetic variants of *BTNL2* and *ANXA11* genes seem to contribute differentially to T-cell sensitization and progression to CBD in Be-exposed individuals (Fig. 1 and Table 1). This functional duality could also be true in sarcoidosis. However it has not been studied due to sarcoidosis heterogeneity, although similar findings have been described for some HLA haplotypes similar associations especially for Löfgren’s syndrome that associate with the risk of disease as well as with its resolution [4].

In summary, this genetic study didn’t demonstrate statistically robust results despite one of the largest cohorts of beryllium-exposed individuals emphasizing the difficulties of genetic studies in rare disease. However, analyses of nominally significant OR for some SNP allow some more detailed hypothesis on the role of some genes relevant for granulomatous diseases.

## Data Availability

Analyzed metadata can be obtained from Karoline I. Gaede on reasonable request in consideration of ethical and legal aspects. Source datasets are subject to the restrictions under which the data were collected and must be requested from the organizations that provided them for this study.

## Abbreviations

ANXA 11: Annexin A11
APC: Antigen presenting cells
BeLPT: Beryllium-specific lymphocyte proliferation test
BeS: Beryllium sensitization
BeEx: Beryllium exposure
BTNL2: Butyrophilin-like 2
CBD: Chronic beryllium disease
CD4+: Cluster of differentiation-4 positive, i.e. T-cells expressing cluster of differentiation-4
CQT: Chi-squared test
HLA: Human leukocyte antigen
HLA-DP: Human leukocyte antigen of the major histocompability complex II (MHC2)
IL23: Interleukin-23
OR: Odd’s ratio
OS9: Osteosarcoma-9
rab 23: Ras-related protein 23
SNP: Single nucleotide polymorphism
US: United States
